# A typical carcinoid tumor of the lung presenting with pure persistent ground-glass opacity on high-resolution computed tomography: a case report

**DOI:** 10.1186/s40792-017-0382-3

**Published:** 2017-10-04

**Authors:** Masafumi Yamaguchi, Fumihiko Hirai, Kenichi Taguchi, Ryo Toyozawa, Makoto Edagawa, Shinichiro Shimamatsu, Kaname Nosaki, Takashi Seto, Mitsuhiro Takenoyama, Yukito Ichinose

**Affiliations:** 1 Department of Thoracic Oncology, National Kyushu Cancer Center, 3-1-1, Notame, Minami-ku, Fukuoka, 811-1395 Japan; 2 Department of Pathology, National Kyushu Cancer Center, 3-1-1, Notame, Minami-ku, Fukuoka, 811-1395 Japan

**Keywords:** Lung typical carcinoid tumor, Pure ground-glass opacity on chest computed tomography

## Abstract

Pure ground-glass opacity nodules (p-GGN) on high-resolution computed tomography (HRCT) generally have been considering typically associated with adenocarcinoma with less invasive nature. We herein reported a patient presenting focal p-GGN on middle lobe of the right lung who underwent surgical resection with its pathological diagnosis turned out to be typical carcinoid tumor.

## Background

Pure ground-glass opacity nodules (p-GGN) are frequently identified in the lung parenchyma on high-resolution computed tomography (HRCT). The pathology of p-GGN on HRCT was reported to be atypical adenomatous hyperplasia (AAH), adenocarcinoma, focal organizing pneumonia/fibrosis [1] and so on. In order to determine a malignant or benign histology, surgical resection is sometimes required.

We herein report a case of a resected typical carcinoid tumor that presented as a persistent p-GGN on HRCT.

## Case presentation

**Fig. 1:**
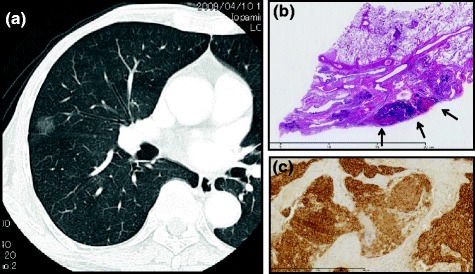
**a** High-resolution computed tomography of the pure ground-glass nodule. Focal p-GGO was identified in the *right middle* lobe of the lung. **b** Hematoxylin staining of the resected specimen. *Small round* to spindle-shaped cells arranged in a spindle cell pattern with finely granular chromatin, inconspicuous nucleoli, and a scant to moderate amount of eosinophilic cytoplasm were seen in the form of many scattered tumor foci (*arrowheads*). **c** Immunohistological staining showed the tumor cells are positive for synaptophysin A

A 74-year-old female was found to have a round p-GGN measuring 12 mm in diameter in the right middle lobe of the lung on chest HRCT, performed for a preoperative work-up of right breast cancer in May 2008. The patient underwent right mastectomy and sentinel lymph node dissection, and the pathological diagnosis was papillotubular carcinoma without lymph node metastasis. Then she was referred to our department in December 2009, and underwent another HRCT examination in March 2010 (Fig. 1a), and there were no significant changes in size of 11 mm in maximal dimension; however, we could not eliminate the possibility of lung adenocarcinoma. Therefore, we performed surgical resection in order to obtain a pathological diagnosis provide treatment simultaneously. FDG-PET was not performed due to the estimated low diagnostic performance of this modality for a diagnosis of p-GGN. The patient underwent video-assisted thoracoscopic middle lobectomy in April 2009. Since the intraoperative frozen pathological diagnosis revealed the tumor to be an adenocarcinoma, additional mediastinal lymphadenectomy was subsequently performed. Histologically, the tumor located just beneath the visceral pleura exhibited the proliferation of small round to spindle-shaped cells arranged in a spindle cell pattern with finely granular chromatin, inconspicuous nucleoli, and a scant to moderate amount of eosinophilic cytoplasm. Necrosis and mitoses were rarely detected (Fig. 1b). Immunohistochemically, the tumor cells were positive for chromogranin A and synaptophysin (Fig. 1c). These features indicated a typical carcinoid tumor of pT1aN0M0.

### Discussion

The term “ground glass opacity (GGO)” is defined as “hazy increased attenuation in the lung that does not obliterate the bronchial and vascular margins” in the lung detected on HRCT, according to the Fleischner Society [2]. This definition is non-specific and does not necessarily reflect the pathological etiology of p-GGN. The presence of p-GGN on HRCT sometimes implies early-stage lung cancer, i.e. adenocarcinoma in situ or so-called bronchioloalveolar adenocarcinoma [3]. With respect to other lung malignancies, Okita et al. reported a rare case of metastatic melanoma in the lung presenting with focal GGO [4]. To our knowledge, this is the first report of a typical carcinoid tumor of the lung that presented with focal p-GGN on HRCT. In general, the appearance of a typical carcinoid tumor on CT is generally a solid nodule [5]. Also, diffuse idiopathic pulmonary neuroendocrine cell hyperplasia (DIPNECH) is now considered to be a precursor for pulmonary carcinoid tumors; however, its appearance on HRCT has generally reported to be diffuse, multifocal GGO due to small airways obstruction evidenced by inspiratory mosaic attenuation and expiratory air trapping [6]. In the present case, the tumor was consisted of many small scattered tumor foci even in the central collapsed area (Fig. 1b); thus, the appearance of the tumor on HRCT might have a possibility to be p-GGN.

## Conclusions

In conclusion, despite its rarity, physicians should consider typical carcinoid tumors in the differential diagnosis of persistent p-GGN on HRCT.
